# Supplementary material for: Characteristic Analysis of Featured Genes Associated with Cholangiocarcinoma Progression
Source: Biomedicines. 2023 Mar 10;11(3):847. doi: 10.3390/biomedicines11030847 (PMC10045321; doi:10.3390/biomedicines11030847)
Supplement: Supplementary file 1 [file biomedicines-11-00847-s001.zip › biomedicines-2182635-supplementary.pdf]

Supplementary data

Characteristic Analysis of Featured Genes Associated Cholangiocarcinoma Progression

Table S1 Clinical characteristic of included patients

A

Submitter_id	Age	Albumin	Ca_199	Cancer_first_degree	Child_pugh	Creatinine	Fetoprotein	Fibrosis _score	Neoplasm_grade	Other_dx	M	N	T	History_of_neoadjuvant_treatment
TCGA-ZH-A8Y2-01A	59	NA	NA	3	NA	NA	7.8	NA	G2	No	M0	NX	T1	No
TCGA-W5-AA30-01A	82	4.3	265	1	NA	1	3.7	No Fibrosis	G3	No	M0	N0	T1	No
TCGA-W5-AA30-11A	82	4.3	265	1	NA	1	3.7	No Fibrosis	G3	No	M0	N0	T1	No
TCGA-W5-AA36-01A	51	4.6	1	NA	A	0.8	14	No Fibrosis	G3	No	M1	N0	T3	Yes
TCGA-W5-AA39-01A	81	3.5	57	2	A	1.1	2.7	No Fibrosis	G4	No	M0	N0	T2	No
TCGA-W5-AA2R-01A	77	4.2	11	3	A	0.9	3.6	No Fibrosis	G3	No	M0	N0	T1	No
TCGA-W5-AA2R-11A	77	4.2	11	3	A	0.9	3.6	No Fibrosis	G3	No	M0	N0	T1	No
TCGA-W5-AA2Z-01A	29	4.6	495	1	A	0.8	1.6	Portal Fibrosis	G3	No	M0	N0	T2	No
TCGA-3X-AAV9-01A	72	2.7	538	2	B	1.1	NA	No Fibrosis	G2	Yes	M0	N0	T1	No
TCGA-W5-AA38-01A	55	4.8	199	1	A	0.6	2.2	Portal Fibrosis	G3	No	M0	N0	T1	No
TCGA-ZH-A8Y4-01A	58	4.2	27	1	NA	0.9	6	NA	G3	Yes	M0	N0	T1	No
TCGA-W5-AA31-01A	71	NA	18	2	NA	0.9	4.4	No Fibrosis	G3	Yes	M0	N0	T1	No
TCGA-W5-AA31-11A	71	NA	18	2	NA	0.9	4.4	No Fibrosis	G3	Yes	M0	N0	T1	No
TCGA-ZH-A8Y5-01A	69	4.3	7	1	NA	1	NA	NA	G3	No	M1	N1	T3	No
TCGA-W5-AA2U-01A	78	4.5	91	1	A	0.8	NA	No Fibrosis	G3	No	M0	N0	T1	No
TCGA-W5-AA2U-11A	78	4.5	91	1	A	0.8	NA	No Fibrosis	G3	No	M0	N0	T1	No
TCGA-4G-AAZO-01A	71	3.8	6	NA	NA	0.9	2.4	Portal Fibrosis	G2	No	M0	N0	T2a	No
TCGA-ZD-A8I3-01A	73	NA	NA	NA	NA		NA	NA	G3	No	M0	N0	T2	No
TCGA-W5-AA2W-01A	31	3.7	104	NA	A	0.6	NA	No Fibrosis	G3	No	M0	N1	T2a	No
TCGA-ZH-A8Y6-01A	41	3.9	29	3	NA	1.2	NA	NA	G2	No	M0	N0	T1	No
TCGA-W5-AA2Q-01A	68	3.7	247	1	A	1	4.3	Portal Fibrosis	G2	No	M0	N0	T2b	No
TCGA-W5-AA2Q-11A	68	3.7	247	1	A	1	4.3	Portal Fibrosis	G2	No	M0	N0	T2b	No
TCGA-ZH-A8Y8-01A	73	NA	39.2	2	NA	0.8	NA	NA	G2	Yes	M0	N0	T1	No
TCGA-W5-AA2I-01A	66	4.4	296	NA	A	1.4	2.9	No Fibrosis	G2	No	M0	N0	T1	No
TCGA-W5-AA2I-11A	66	4.4	296	NA	A	1.4	2.9	No Fibrosis	G2	No	M0	N0	T1	No

TCGA-W5-AA2H-01A	70	4.3	44	2	A	0.9	3.1	No Fibrosis	G3	No	M0	N0	T3	No
TCGA-W5-AA2X-01A	67	4	203	1	A	0.8	4	Portal Fibrosis	G4	No	M1	N1	T2b	No
TCGA-W5-AA2X-11A	67	4	203	1	A	0.8	4	Portal Fibrosis	G4	No	M1	N1	T2b	No
TCGA-3X-AAVA-01A	50	NA	8	NA	NA	0.5	5.4	Portal Fibrosis	G2	No	M0	NX	T2b	No
TCGA-3X-AAVE-01A	60	4	NA	NA	A	0.9	3.6	Fibrous Speta	G2	No	M0	N0	T2	No
TCGA-W5-AA2T-01A	64	4.4	33	NA	A	0.6	NA	No Fibrosis	G3	Yes	M0	N0	T2	No
TCGA-4G-AAZT-01A	62	4.4	52.8	NA	NA	0.6	2	Portal Fibrosis	G2	No	M0	N0	T1	No
TCGA-W6-AA0S-01A	46	4	NA	NA	A	0.6	NA	Portal Fibrosis	G2	No	MX	N0	T1	No
TCGA-W5-AA33-01A	60	4.4	17	2	A	1	2	No Fibrosis	G3	No	M0	N0	T1	No
TCGA-W5-AA2G-01A	62	4.1	71	1	A	0.7	2.5	No Fibrosis	G3	No	M0	N0	T1	No
TCGA-W5-AA34-01A	75	4.2	25	4	A	0.6	2.5	No Fibrosis	G2	No	M0	N0	T1	No
TCGA-W5-AA34-11A	75	4.2	25	4	A	0.6	2.5	No Fibrosis	G2	No	M0	N0	T1	No
TCGA-3X-AAVB-01A	70	4.3	322	NA	A	0.9	NA	NA	G1	No	M1	N1	T3	No
TCGA-W5-AA2O-01A	57	4.1	34	NA	A	1.1	3.5	No Fibrosis	G3	No	M0	N0	T1	No
TCGA-ZU-A8S4-01A	52	4.2	115	NA	NA	0.9	1.9	No Fibrosis	G3	No	MX	NX	T1	No
TCGA-ZU-A8S4-11A	52	4.2	115	NA	NA	0.9	1.9	No Fibrosis	G3	No	MX	NX	T1	No
TCGA-YR-A95A-01A	52	4.3	18.15	NA	NA	0.8	1.38	NA	G2	No	M1	NX	T2	No
TCGA-3X-AAVC-01A	72	2.4	NA	NA	B	0.8	NA	Portal Fibrosis	G3	No	M0	N0	T1	NO
TCGA-WD-A7RX-01A	71	3.7	34	1	NA	0.9	2.6	Fibrous Speta	G2	No	MX	NX	T2b	NA
TCGA-ZH-A8Y1-01A	74	4.6	6910	NA	NA	0.8	NA	NA	G2	Yes	M0	N1	T3	NA

B

Submitter_id	Neoplasm_ cancer_status	Platelet_ result	Prothromb in_time	Family_can cer_history	Vascular_tumor_c ell_type	Days_to_ death.	Gender	Race	Vital_status	Prior_malignan cy.diagnose	Prior_treat ment	Tumor_stage.di agnose	BMI
TCGA-ZH-A8Y2-01A	NA	NA	NA	Yes	Micro	701	Female	Black	Dead	No	No	Stage i	26.72
TCGA-W5-AA30-01A	Tumor free	134	1	Yes	None	NA	Male	White	Alive	No	No	Stage i	28.83
TCGA-W5-AA30-11A	Tumor free	134	1	Yes	None	NA	Male	White	Alive	No	No	Stage i	28.81
TCGA-W5-AA36-01A	NA	197	1.3	NA	None	1402	Female	White	Dead	No	Yes	Stage iv	24.97
TCGA-W5-AA39-01A	NA	350	1.2	Yes	None	170	Male	White	Dead	No	No	Stage ii	25.82
TCGA-W5-AA2R-01A	Tumor free	272	0.8	Yes	None	NA	Female	White	Alive	No	No	Stage i	28.84
TCGA-W5-AA2R-11A	Tumor free	272	0.8	Yes	None	NA	Female	White	Alive	No	No	Stage i	28.84
TCGA-W5-AA2Z-01A	Tumor free	242	1	Yes	None	NA	Female	White	Alive	No	No	Stage ii	31.24
TCGA-3X-AAV9-01A	NA	254	NA	Yes	None	339	Male	Asian	Dead	Yes	No	Stage i	18.20
TCGA-W5-AA38-01A	TUMOR FREE	239	1	Yes	None	NA	Female	White	Alive	No	No	Stage i	25.86
TCGA-ZH-A8Y4-01A	NA	208	1.1	Yes	None	741	Male	White	Dead	Yes	No	Stage i	26.27
TCGA-W5-AA31-01A	Tumor free	151	1.1	Yes	None	NA	Male	White	Alive	Yes	No	Stage i	31.01
TCGA-W5-AA31-11A	Tumor free	151	1.1	Yes	None	NA	Male	White	Alive	Yes	No	Stage i	31.01
TCGA-ZH-A8Y5-01A	With tumor	269	1	Yes	Micro	NA	Male	White	Alive	No	No	Stage ivb	27.75
TCGA-W5-AA2U-01A	NA	305	1	Yes	None	627	Female	White	Dead	No	No	Stage i	24.65
TCGA-W5-AA2U-11A	NA	305	1	Yes	None	627	Female	White	Dead	No	No	Stage i	24.65
TCGA-4G-AAZO-01A	NA	164	11.6	No	None	NA	Female	White	Alive	No	No	Stage ii	25.84
TCGA-ZD-A8I3-01A	With tumor	NA	NA	No	NA	169	Female	Asian	Dead	No	No	Stage Ii	21.20
TCGA-W5-AA2W-01A	NA	217	1.1	No	None	924	Female	White	Dead	No	No	Stage iva	32.62
TCGA-ZH-A8Y6-01A	Tumor free	279	1	Yes	None	NA	Female	Black	Alive	No	No	Stage i	38.52
TCGA-W5-AA2Q-01A	Tumor free	174	9.3	Yes	None	NA	Male	White	Alive	No	No	Stage ii	24.61
TCGA-W5-AA2Q-11A	Tumor free	174	9.3	Yes	None	NA	Male	White	Alive	No	No	Stage ii	24.61
TCGA-ZH-A8Y8-01A	Tumor free	261	1.1	Yes	None	NA	Male	White	Alive	Yes	No	Stage i	29.26
TCGA-W5-AA2I-01A	NA	138	1.1	No	None	1939	Male	White	Dead	No	No	Stage i	39.45
TCGA-W5-AA2I-11A	NA	138	1.1	No	None	1939	Male	White	Dead	No	No	Stage i	39.45
TCGA-W5-AA2H-01A	NA	369	0.9	Yes	None	NA	Female	White	Alive	No	No	Stage iii	27.05
TCGA-W5-AA2X-01A	NA	541	1	Yes	Micro	271	Male	White	Dead	No	No	Stage ivb	31.55

TCGA-W5-AA2X-11A	NA	541	1	Yes	Micro	271	Male	White	Dead	No	No	Stage ivb	31.55
TCGA-3X-AAVA-01A	NA	214	NA	No	None	445	Female	White	Dead	No	No	Stage ii	NA
TCGA-3X-AAVE-01A	Tumor free	233	0.9	NA	Micro	NA	Male	Asian	Alive	No	No	Stage ii	23.04
TCGA-W5-AA2T-01A	NA	274	1	No	None	1220	Female	White	Dead	Yes	No	Stage ii	25.81
TCGA-4G-AAZT-01A	NA	354	11.7	No	None	NA	Male	White	Alive	No	No	Stage i	21.22
TCGA-W6-AA0S-01A	With tumor	267	1	No	None	NA	Female	White	Alive	No	No	Stage i	29.42
TCGA-W5-AA33-01A	NA	231	1.1	Yes	None	NA	Male	White	Alive	No	No	Stage i	34.72
TCGA-W5-AA2G-01A	Tumor free	159	1.2	Yes	None	NA	Female	White	Alive	No	No	Stage i	20.56
TCGA-W5-AA34-01A	With tumor	203	1	Yes	None	555	Female	White	Dead	No	No	Stage i	27.73
TCGA-W5-AA34-11A	With tumor	203	1	Yes	None	555	Female	White	Dead	No	No	Stage i	27.73
TCGA-3X-AAVB-01A	NA	215	1.1	No	None	NA	Female	White	Alive	No	No	Stage ivb	26.29
TCGA-W5-AA2O-01A	NA	150	0.8	No	None	640	Male	White	Dead	No	No	Stage i	26.17
TCGA-ZU-A8S4-01A	NA	279	1.1	No	None	98	Male	White	Dead	No	No	Stage i	36.43
TCGA-ZU-A8S4-11A	NA	279	1.1	No	None	98	Male	White	Dead	No	No	Stage i	36.42
TCGA-YR-A95A-01A	NA	240	0.9	Yes	NA	26	Male	White	Dead	No	No	Stage iv	24.57
TCGA-3X-AAVC-01A	Tumor free	255	1.1	NA	None	NA	Female	White	Alive	No	No	Stage i	41.13
TCGA-WD-A7RX-01A	NA	156	12.2	Yes	None	21	Female	White	Dead	No	No	Stage ii	23.60
TCGA-ZH-A8Y1-01A	NA	272	0.9	No	Micro	385	Female	White	Dead	Yes	No	Stage iva	28.65

Abbreviations: NA: Not applicable; BMI: Body Mass Index; M: Stage M; N: Stage N; T: Stage T.

Table S2 Top five gene ontology (GO) analysis of differentially expressed genes (DEGs)

ID	Description	GeneRatio	BgRatio	pvalue	p.adjust	qvalue	Count
GO:0004497	Monooxygenase activity	33/1270	99/17696	2.27E-14	9.88E-12	8.09E-12	33
GO:0022803	Passive transmembrane transporter activity	83/1270	475/17696	2.30E-14	9.88E-12	8.09E-12	83
GO:0046873	Metal ion transmembrane transporter activity	78/1270	439/17696	5.75E-14	1.26E-11	1.03E-11	78
GO:0015267	Channel activity	82/1270	474/17696	5.87E-14	1.26E-11	1.03E-11	82
GO:0005216	Ion channel activity	76/1270	435/17696	2.99E-13	5.14E-11	4.21E-11	76

Table S3 Top five KEGG analysis of DEGs

ID	Description	GeneRatio	BgRatio	pvalue	p.adjust	qvalue	Count
hsa04080	Neuroactive ligand-receptor interaction	72/586	341/8080	2.64E-17	7.68E-15	6.44E-15	72
hsa05204	Chemical carcinogenesis	31/586	83/8080	3.64E-15	5.29E-13	4.44E-13	31
hsa00982	Drug metabolism - cytochrome P450	27/586	72/8080	2.02E-13	1.88E-11	1.57E-11	27
hsa00980	Metabolism of xenobiotics by cytochrome P450	28/586	78/8080	2.58E-13	1.88E-11	1.57E-11	28
hsa00140	Steroid hormone biosynthesis	24/586	61/8080	1.32E-12	7.66E-11	6.43E-11	24

Table S4 Power table of k soft threshold

Power	SFT.R.sq	Slope	Truncated.R.sq	Mean.k.	Median.k.	Max.k.
1	0.262156	4.862341	0.476511	255.7166	257.4568	360.6233
2	0.320514	3.667307	0.127644	77.46597	75.13232	135.3579
3	0.308174	0.907199	0.930445	33.36389	28.52398	75.85615
4	0.615427	-0.986000	0.865914	18.19474	12.91985	59.52628
5	0.897760	-1.296660	0.960372	11.57460	6.765432	50.36065
6	0.899009	-1.349250	0.925069	8.141229	3.796767	44.29117
7	0.868399	-1.368220	0.889772	6.125363	2.355469	39.88780
8	0.877368	-1.328590	0.897098	4.830520	1.482092	36.51241
9	0.822534	-1.354760	0.829417	3.941776	0.997818	33.84368
10	0.882800	-1.312250	0.897619	3.300356	0.682807	31.64898
11	0.917943	-1.294630	0.930072	2.446463	0.341695	28.21161
12	0.952010	-1.263250	0.961197	1.911127	0.177570	25.59922
13	0.915174	-1.285350	0.920802	1.548228	0.100136	23.51476
14	0.952819	-1.239650	0.960856	1.288135	0.057451	21.79482
15	0.952677	-1.225270	0.955693	1.093827	0.033978	20.34159

Table S5 Summary of the genes in blue and magenta module

**Blue module**						
AKNAD1	CES5A	DLX4	FRMPD2	MELK	RAD54L	STRC
AMDHD1	CHRNG	E2F7	FUT5	MND1	RDM1	TICRR
APOC2	CLSPN	E2F8	GOLGA6B	MTFR2	RGS17	TMEM61
BUB1	CLVS1	ERCC6L	GPR19	NEIL3	RIBC2	TRIM72
BUB1B	CNFN	EXO1	HGFAC	NEK2	SHCBP1	TRPV5
C20orf144	CTSV	FALEC	HJURP	NOS1	SIX2	ULBP1
CDC25C	CYP2A13	FAM227A	HOXA6	NUF2	SKA1	ZNF695
CDCA2	DDX11-AS1	FAM81B	HS6ST3	PBK	SKA3	
CDKN2B-AS1	DEPDC1	FANCB	KIF18A	PCDHB8	SLC22A24	
CENPA	DIAPH3	FBXO43	KIF18B	PCP4L1	SLC5A7	
CENPM	DLGAP5	FNDC8	LRRC36	PPP1R1A	SMIM23	
CEP55	DLX1	FOXD3-AS1	MCM10	PRPH	SPC25	
**magenta module**						
ADAMTS18	CLCNKA	FRMD5	IGDCC3	MIR4326	PROZ	TBX10
ADH7	CLEC1B	FSTL4	IGF1	MIR6797	PRR5-ARHGAP8	TBX4
ALPP	CPSF4L	FXYD3	IGF2BP1	MSX2	PTGES3L	TDGF1
ARHGAP8	CRHBP	FXYD4	IGFL4	MUC2	PTGES3L-AARSD1	TECTB
ASB4	CYP2D6	FZD10	IRX2	MYB	PZP	TERT
ASCL5	CYP2W1	GABRQ	KCNV2	MYO18B	RAD51AP2	TH
ASIC4	DEFA3	GABRR1	KHDC1	MYT1	RASSF10	TRH
ASPG	DNAH9	GAD1	KLHL35	NECAB2	RBBP8NL	TRPC5
BDKRB1	DRD2	GAL	KLK12	NOTUM	RD3L	TRPM5
BEST3	DRP2	GBX2	KLK14	NPFFR1	RFPL1	TUBB8
BHLHA9	DSC3	GDF2	KLK6	OTOGL	RFX4	VSIG8
BMP7	ECEL1	GJB7	KRT6B	OTX1	RNFT2	VSNL1
C10orf95	ELAVL2	GLYATL3	LEMD1	PAK6	RPS10-NUDT3	WIF1
C2CD4D	ELF5	GNG13	LGALS9B	PCP4	SCARA5	XKR6
CALB2	ENPP3	GNG4	LINC00511	PDIA2	SH3GL3	ZIC1
CALML3	EPHA10	GREM2	LINC01535	PGC	SHISA6	ZIC4
CASKIN1	EPN3	HAL	LINC01572	PHEX	SLC25A52	ZNF280B
CCL24	EVPLL	HOTAIR	LIPI	PITX2	SLC30A3	ZNF750
CCNI2	EVX1	HOXB13	LIX1	PKP1	SLC35G5	
CD164L2	FAM24B	HOXC13	LOXL1-AS1	PLA2G4D	SLC6A14	
CDCA7	FAM72C	HOXC5	LRRTM1	PLSCR2	SPDYE2	
CDX2	FAM72D	HOXC6	LY6G6C	PNCK	STRA6	
CHRNA4	FGF11	HOXC8	LYPD1	PRAC2	TAS2R31	
CHRND	FGF9	HSF2BP	MFSD6L	PRKCG	TAS2R5	

Table S6 Summary of the genes in biological process in GO term

Category	Term	adj_pval	Genes
BP	chromosome segregation	1.30E-05	CDCA2, HJURP, NEK2, SKA3, SKA1
BP	mitotic sister chromatid segregation	3.97E-05	KIF18A, KIF18B, NEK2, CENPA
BP	condensed chromosome outer kinetochore	1.75E-04	BUB1B, SKA3, SKA1
BP	mitotic nuclear division	0.005101	NUF2, SKA3, SKA1
BP	regulation of microtubule polymerization or depolymerization	0.021272	SKA3, SKA1
BP	regulation of dopamine secretion	0.028264	CHRNA4, DRD2
BP	exploration behavior	0.031742	CHRNA4, LRRTM1
BP	microtubule depolymerization	0.035208	KIF18A, KIF18B
BP	mitotic spindle assembly checkpoint	0.042102	BUB1B, BUB1
BP	patterning of blood vessels	0.082472	GBX2, PITX2

Table S7 Overall survival in related with 26 hub genes

Genes	Cutoff value of TPM	The number of patient（High/Low）	LogRank P value
VSNL1	8512.371216	22/14	0.027
TICRR	22172.12094	18/18	0.017
TH	3037.758318	16/20	0.045
TECTB	11904.19283	18/18	0.044
TBX4	7167.037324	20/16	0.011
SKA3	24504.97445	18/18	0.034
SKA1	24504.97445	18/18	0.031
RAD51AP2	9398.972322	18/18	0.048
PTGES3L	21005.79272	18/18	0.040
PITX2	6824.276851	18/18	0.048
PBK	25450.77026	18/18	0.040
NEK2	26870.38470	18/18	0.029
MUC2	4780.971316	18/18	0.036
MND1	24934.93430	16/20	0.032
MELK	26336.19784	18/18	0.028
KIF18B	25680.08727	18/18	0.015
IGDCC3	4985.554888	18/18	0.049
HJURP	25966.18768	18/18	0.020
FGF11	18462.29435	16/20	0.028
EXO1	28219.92744	18/18	0.022
DEPDC1	21002.04838	16/20	0.018
CDC25C	26269.01426	18/18	0.017
BUB1B	26406.15473	20/16	0.040
BUB1	27445.95063	18/18	0.020
ASPG	3996.028732	18/18	0.025
ALPP	3512.083203	18/18	0.049

Abbreviations: TPM: transcripts per million.

Table S8 The receiver operating characteristic (ROC) curve of 61genes

Genes	ROC value	Genes	ROC value
VSNL1	AUC=0.972	LIX1	AUC=0.819
VSIG8	AUC=0.910	KIF18B	AUC=1.000
TRPM5	AUC=0.775	KIF18A	AUC=1.000
TRPC5	AUC=0.991	IGFL4	AUC=0.917
TICRR	AUC=0.991	IGDCC3	AUC=0.855
TH	AUC=0.917	HJURP	AUC=1.000
TECTB	AUC=0.901	GLYATL3	AUC=0.972
TDGF1	AUC=0.895	GJB7	AUC=0.870
TBX4	AUC=0.932	GBX2	AUC=0.793
SLC30A3	AUC=0.935	FZD10	AUC=0.981
SKA3	AUC=1.000	FSTL4	AUC=0.860
SKA1	AUC=1.000	FGF11	AUC=0.923
SH3GL3	AUC=0.835	EXO1	AUC=1.000
RD3L	AUC=0.929	ERCC6L	AUC=1.000
RAD54L	AUC=1.000	ENPP3	AUC=0.957
RAD51AP2	AUC=0.779	DRD2	AUC=0.832
PTGES3L-AARSD1	AUC=0.835	DLGAP5	AUC=0.997
PTGES3L	AUC=0.994	DEPDC1	AUC=1.000
PITX2	AUC=0.824	CLEC1B	AUC=1.000
PCP4	AUC=0.802	CHRNA4	AUC=1.000
PBK	AUC=1.000	CEP55	AUC=1.000
NUF2	AUC=0.997	CENPA	AUC=1.000
NOTUM	AUC=0.932	CDCA2	AUC=1.000
NEK2	AUC=1.000	CDC25C	AUC=1.000
MYB	AUC=0.957	CALML3	AUC=0.954
MUC2	AUC=0.852	BUB1B	AUC=1.000
MND1	AUC=1.000	BUB1	AUC=1.000
MELK	AUC=1.000	BEST3	AUC=0.971
MCM10	AUC=0.997	ASPG	AUC=1.000
LY6G6C	AUC=0.935	ALPP	AUC=0.809
LRRTM1	AUC=0.812		

Table S9 The receiver operating characteristic (ROC) curve of 10 hub genes

Genes	ROC value
VSNL1	0.810
TRPM5	0.799
TH	0.779
PCP4	0.789
IGDCC3	0.789
RAD51AP2	0.735
NOTUM	0.714
MUC2	0.742
BUB1B	0.721
BUB1	0.714

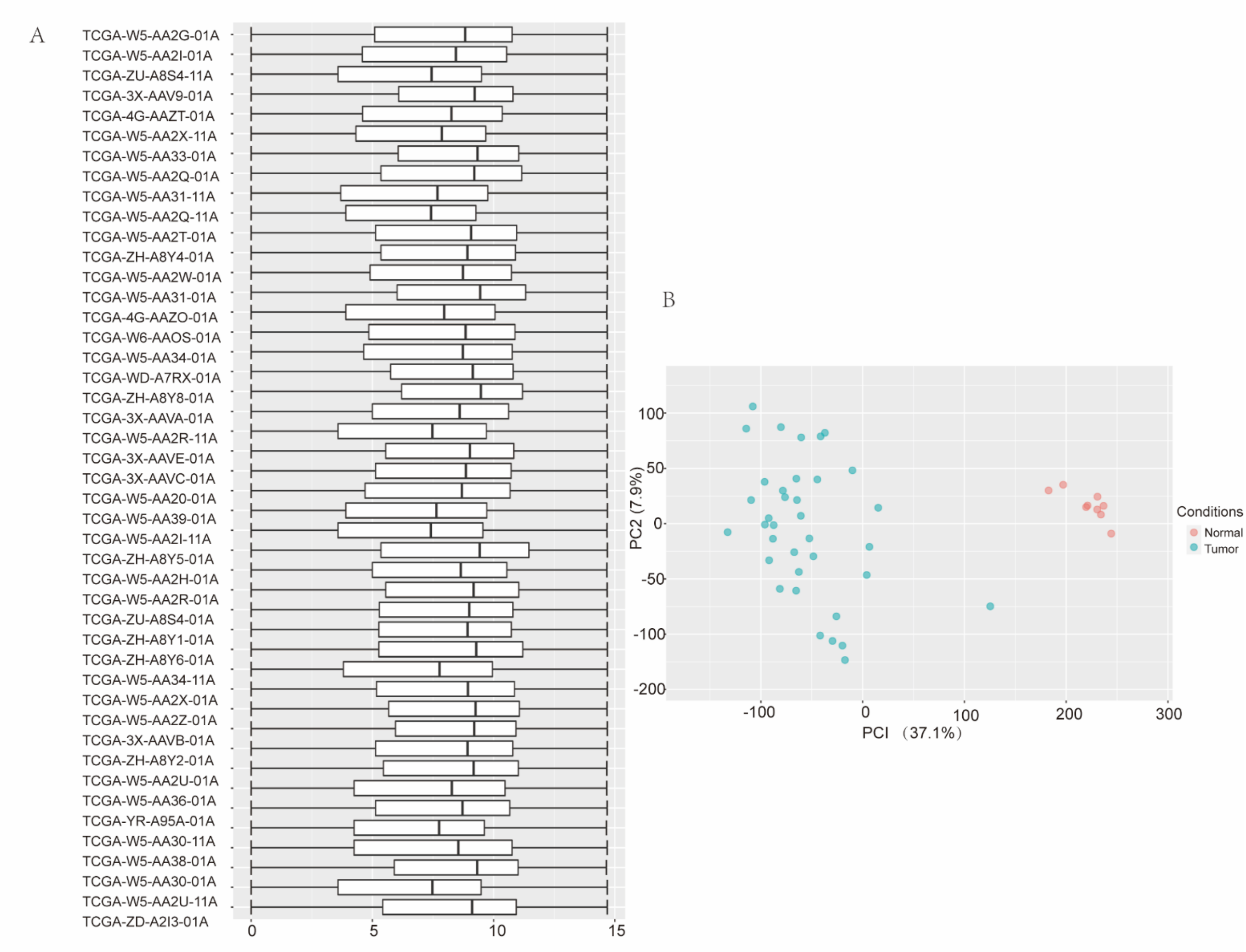

Figure S1. Data before normalization. (A) Box plot of 45 sample before Normalization. (B) Scatter plot of 45 sample before Normalization.

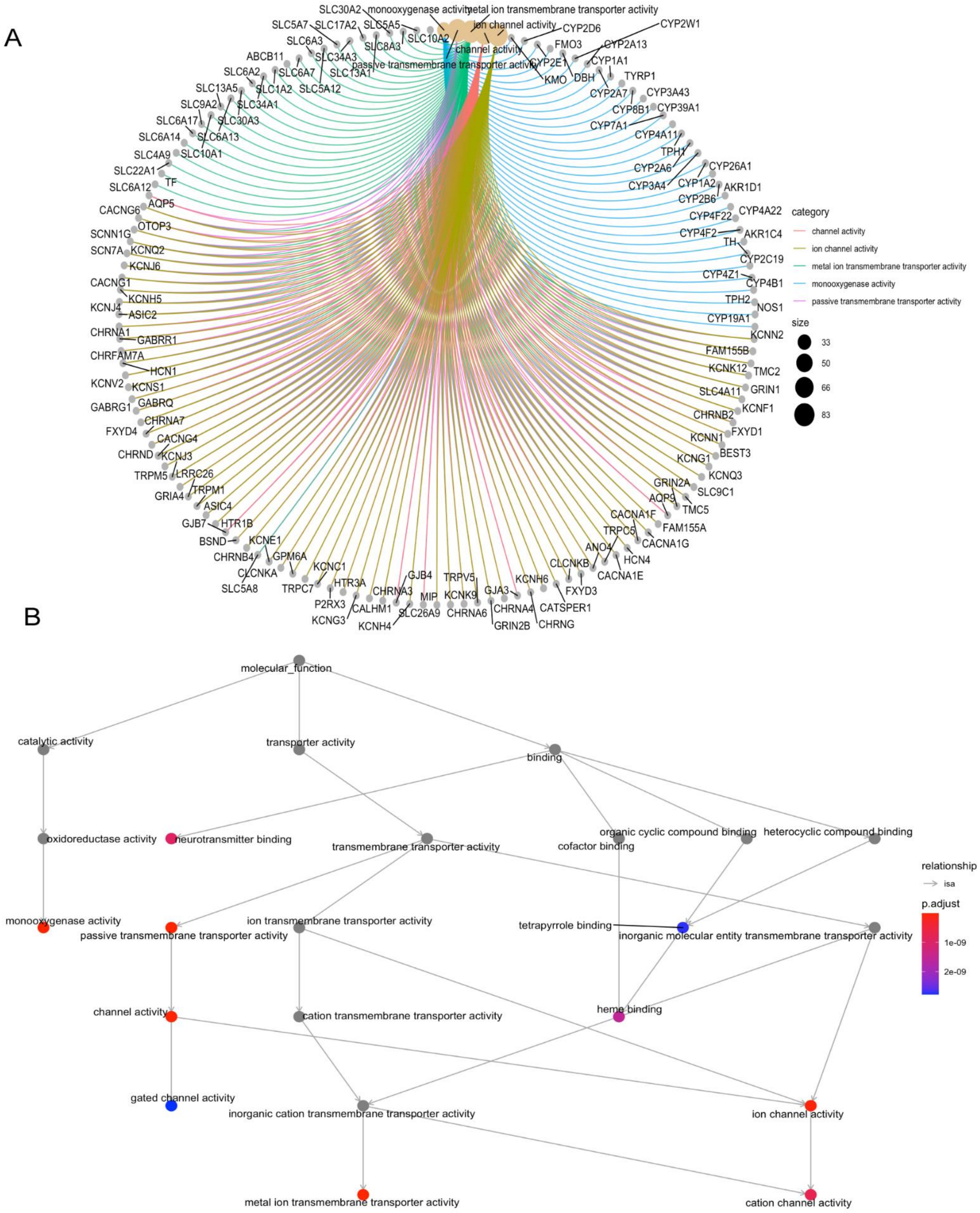

Figure S2. DEGs annotation. (A) Concept network of top 5 GO analysis (B) Molecular function of concept network of GO analysis

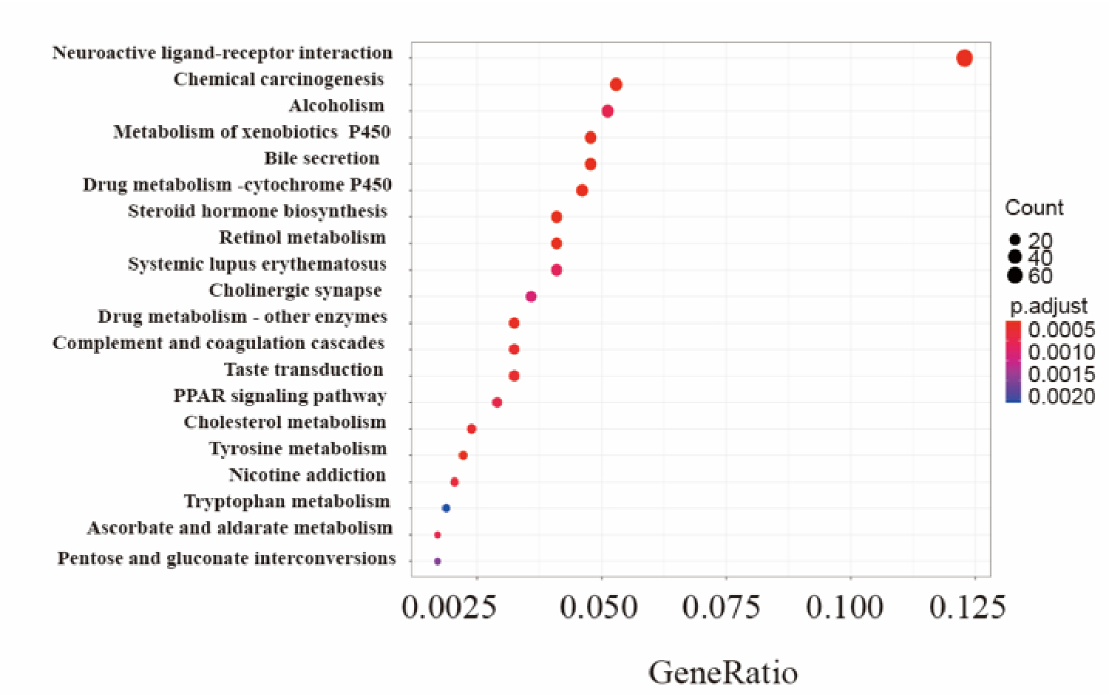

Figure S3. Functional annotation of DEGs. Bubble diagram of significant difference KEGG pathway.

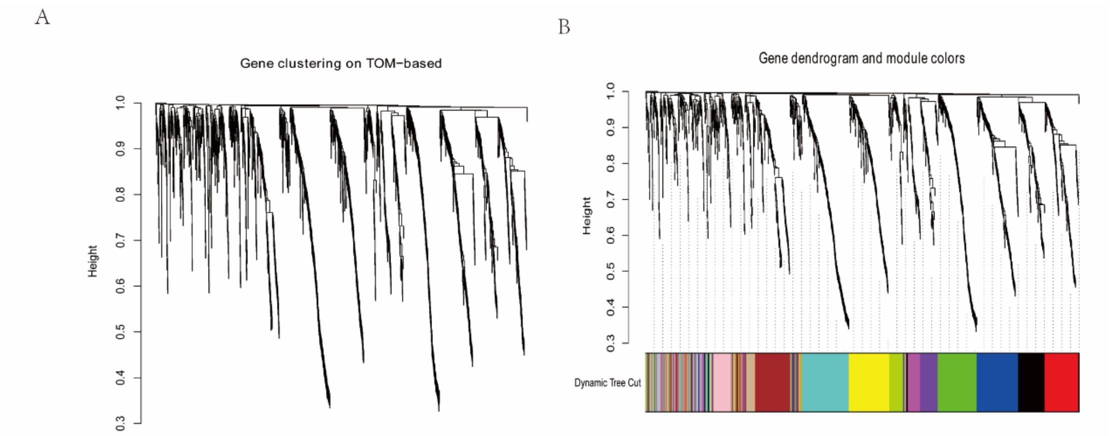

Figure S4. WGCNA analysis. (A) The plot of TOM-based hierarchical gene clustering tree. (B) Dynamic tree cut was used to identify modules (the minimum module size was defined as 30, and deepSplit = 2).

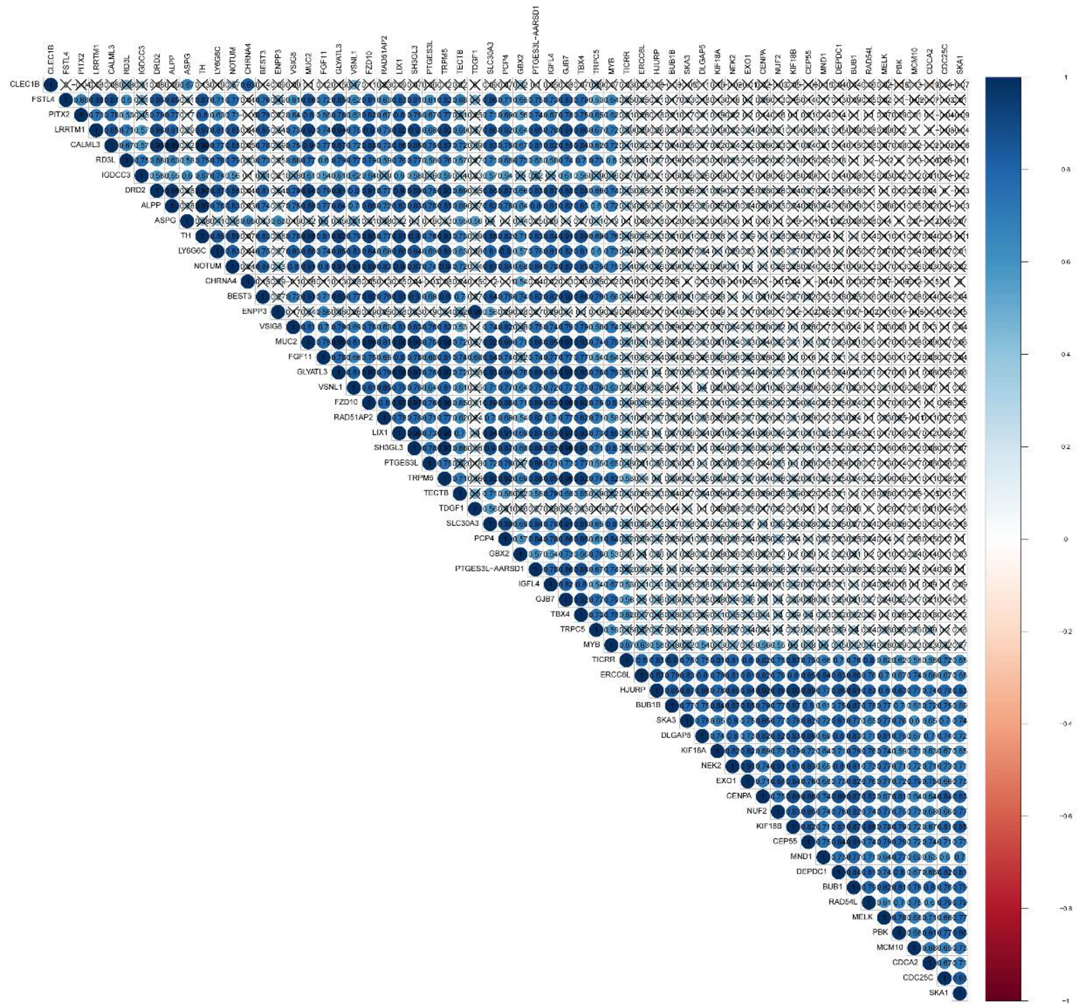

Figure S5. Co-expression heatmap of 61 genes with annotated coefficients. The higher the positive correlation, the darker the blue, the higher the negative correlation, and the darker the red.

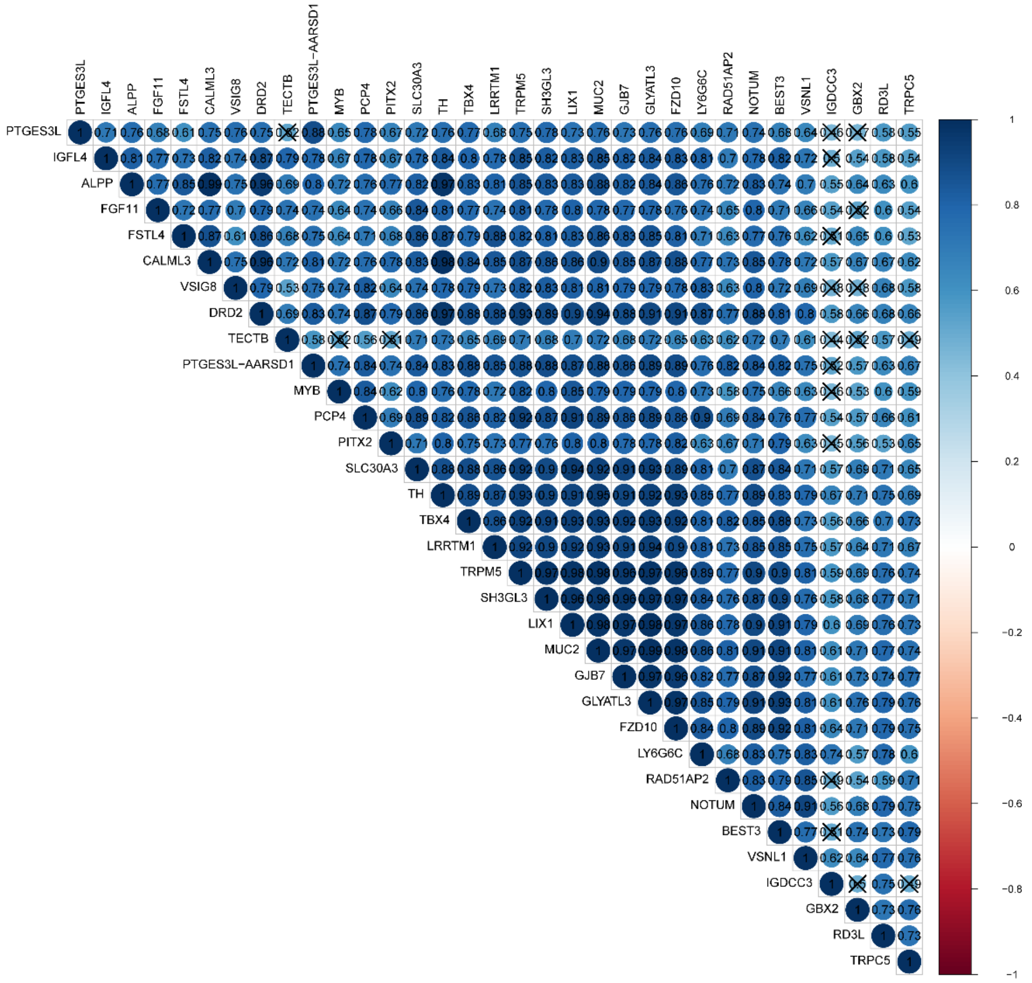

Figure S6. Subgroup of co-expression heatmap of 33 hub gene with coefficients annotated. The higher the positive correlation, the darker the blue, the higher the negative correlation, and the darker the red.

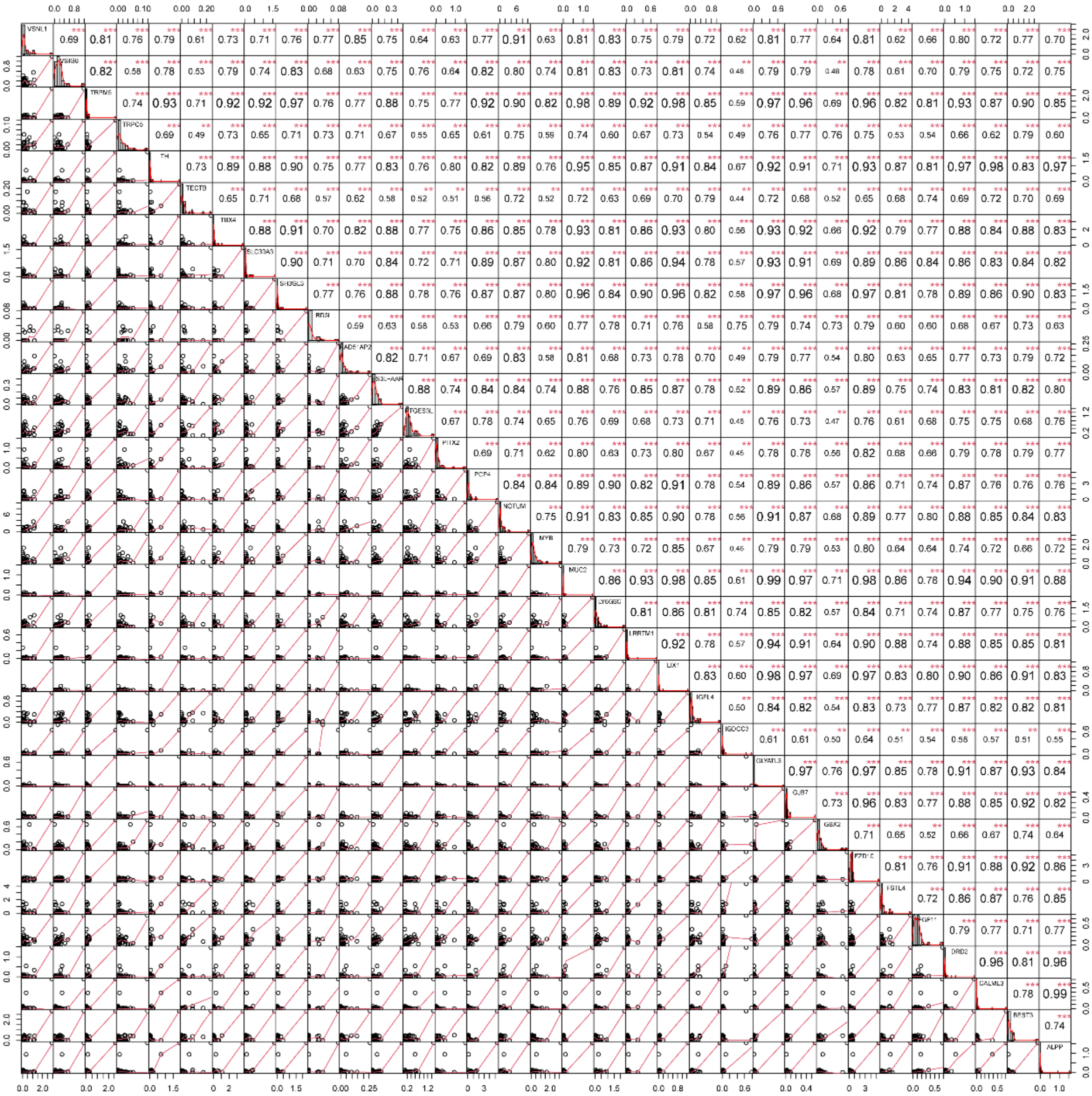

Figure S7. Subgroup of co-expression dotplot of 23 hub gene with coefficients annotated.

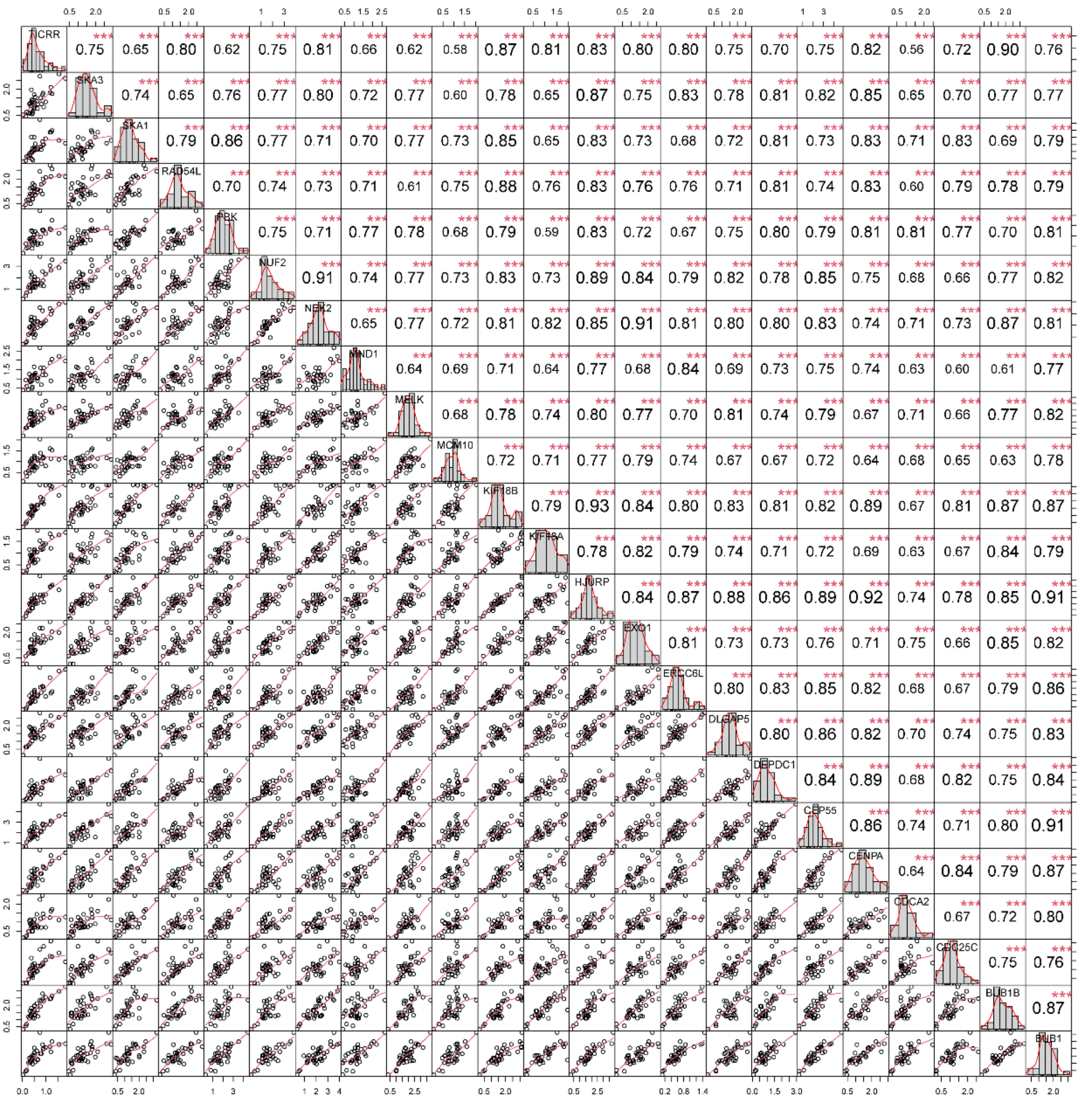

Figure S8. Subgroup of co-expression dotplot of 23 hub gene with coefficients annotated.

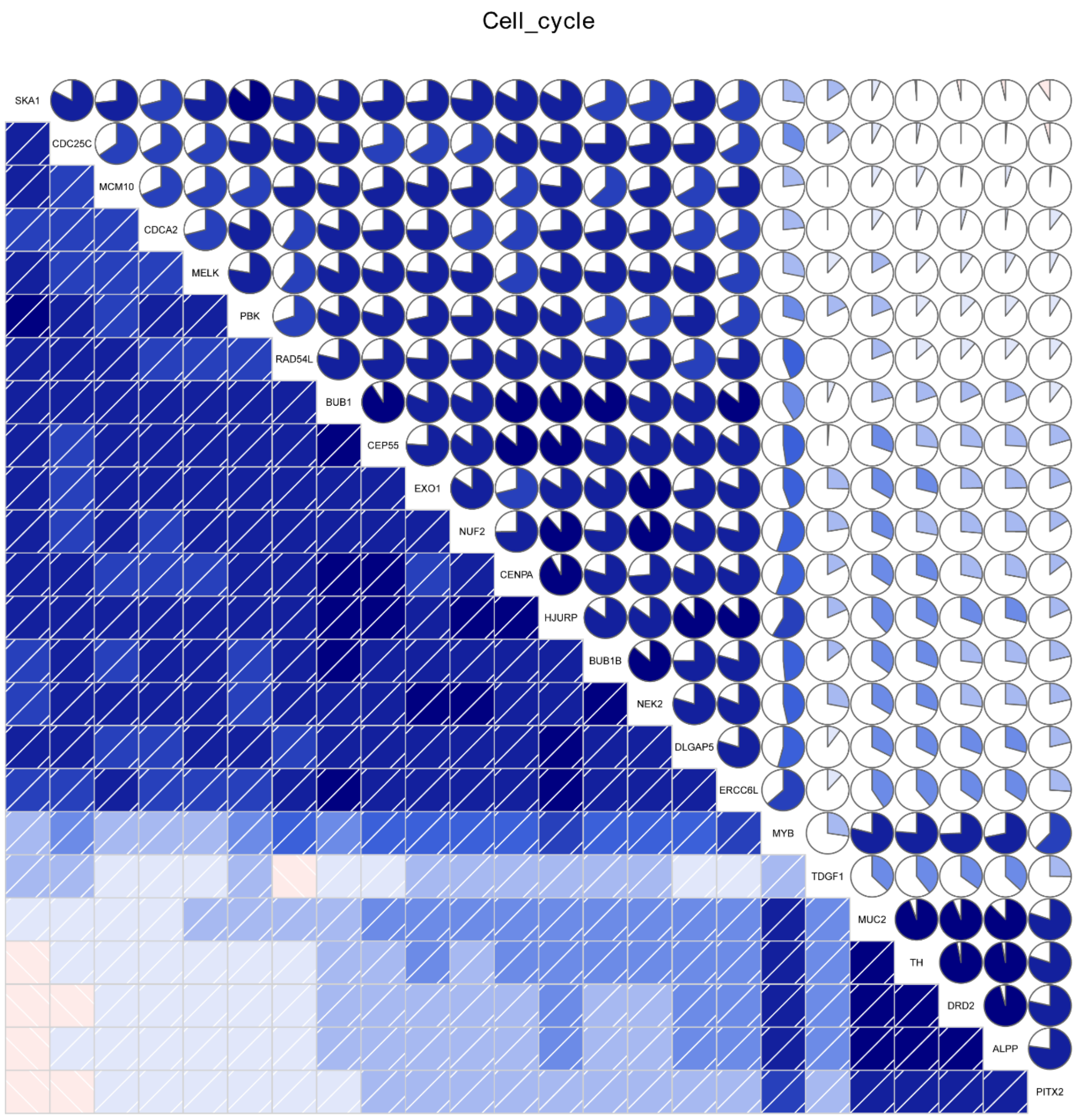

Figure S9. Heatmap of cell cycle, blue means high value. The higher the positive correlation, the darker the blue, the higher the negative correlation, and the darker the red.

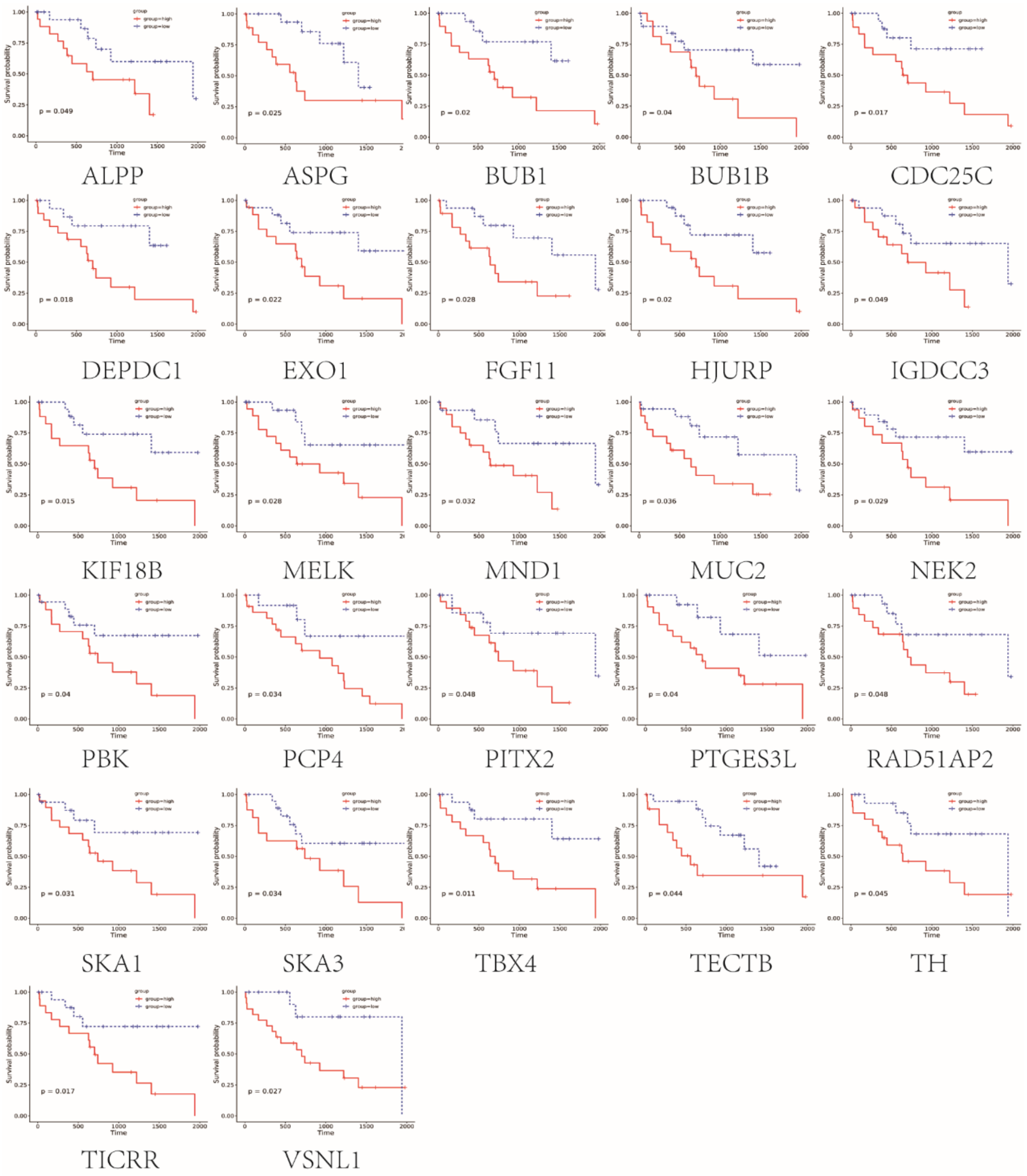

Figure S10. Kaplan–Meier survival curves for the 27 hub genes related to overall survival for 36 patients with CCA based on the TCGA CCA cohort. Blue curve was low expression group, red curve was high expression group, and all genes $P < 0.05$.

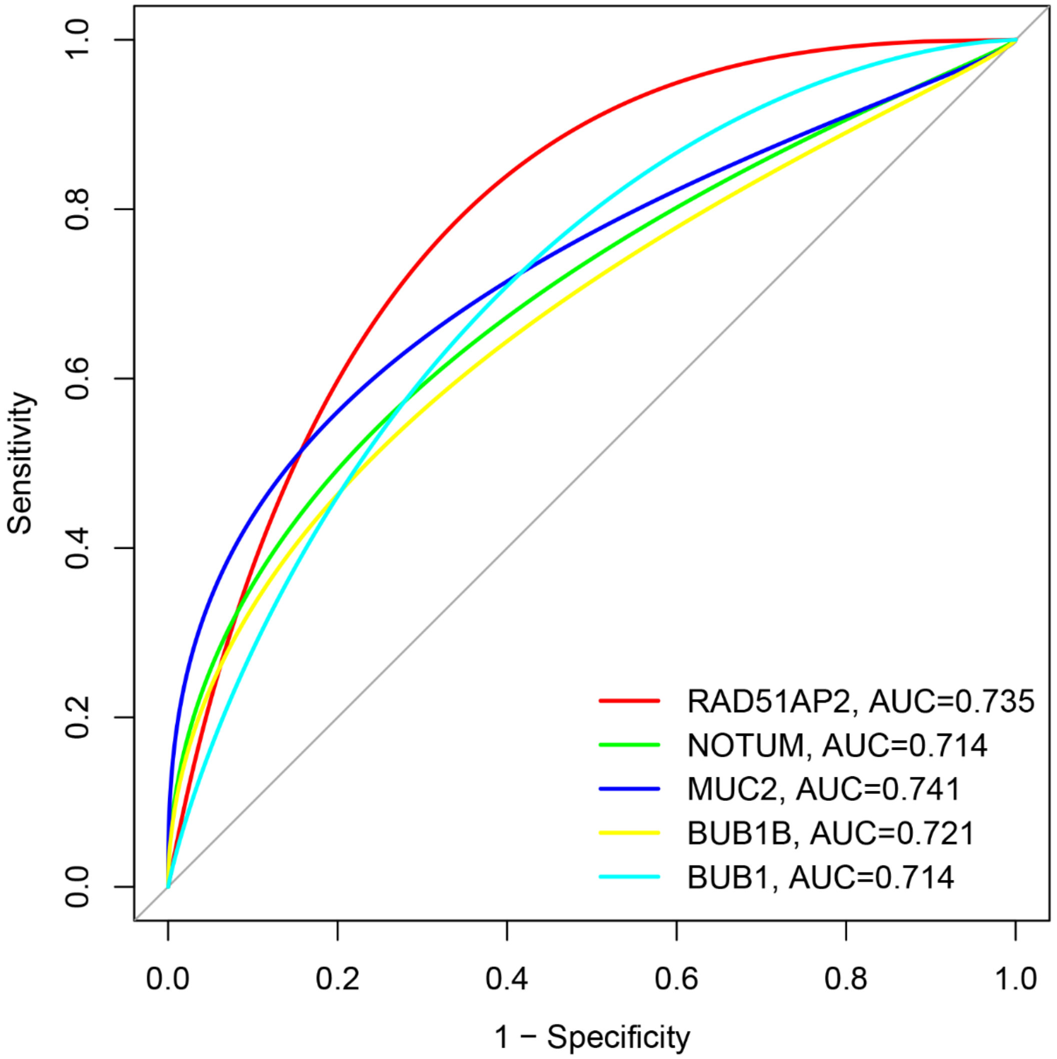

Figure S11. The receiver operating characteristic (ROC) curve verified that 5 hub genes could be employed to distinguish tumorigenic progression level of CCA. All genes AUC > 0.7

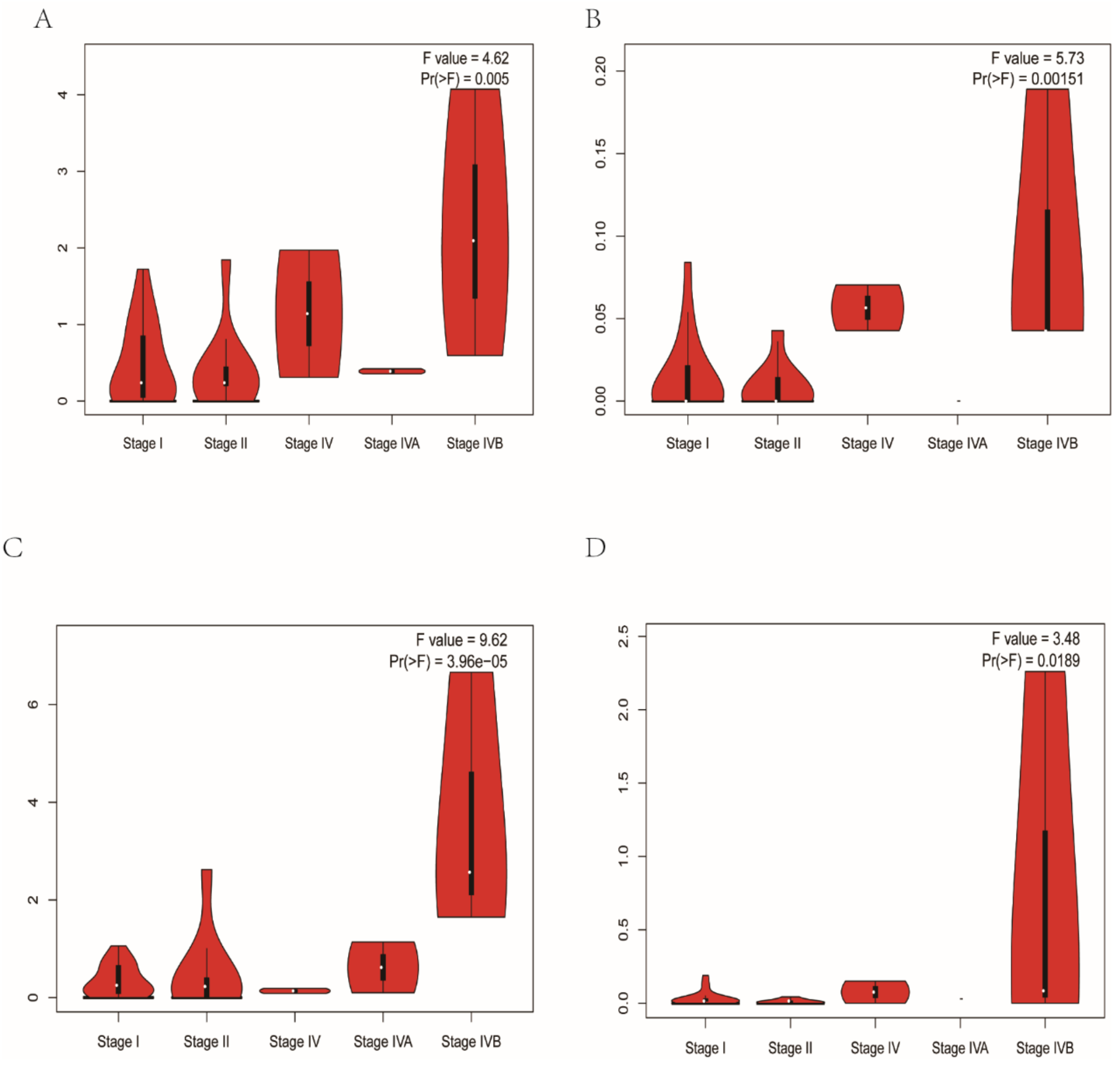

Figure S12. The tumorigenic progression by stage box plots of (A) VSNL1, (B) RAD51AP2, (C) PCP4, (D) MUC2.
